# Fungal Community Ecology Using MALDI-TOF MS Demands Curated Mass Spectral Databases

**DOI:** 10.3389/fmicb.2019.00315

**Published:** 2019-02-27

**Authors:** Matheus Sanitá Lima, Rosymar Coutinho de Lucas, Nelson Lima, Maria de Lourdes Teixeira de Moraes Polizeli, Cledir Santos

**Affiliations:** ^1^Department of Biology, University of Western Ontario, London, ON, Canada; ^2^Biology Department, Faculty of Philosophy, Sciences and Letters of Ribeirão Preto, University of São Paulo, Ribeirão Preto, Brazil; ^3^CEB – Biological Engineering Centre, University of Minho, Braga, Portugal; ^4^Department of Chemical Science and Natural Resources, BIOREN-UFRO, Universidad de La Frontera, Temuco, Chile

**Keywords:** MALDI-TOF MS, fungal community ecology, fungal biodiversity, microbial Biological Resource Centers, mass spectral databases, culturomics, eukaryotic microbial communities, high-throughput species identification

Fungi and bacteria are the main terrestrial decomposers as they act in virtually all ecological niches (Ivarsson et al., 2016). Fungi are also widely used in human activities in the production of beverages, food, and high-value biotechnological molecules such as enzymes, pigments, vitamins, and antibiotics (Polizeli et al., 2005; Narsing Rao et al., 2017). Beyond that, fungi are model organisms for basic and applied research from genetics to ecology (dos Santos Castro et al., 2016; Peay et al., 2016). However, they can also be a threat as many of them are pathogens of plants, invertebrates, humans, and other vertebrates (Arvanitis et al., 2013; Hohl, 2014; Peay et al., 2016). Undoubtedly, rapid and accurate identification of fungi is fundamentally important.

Megadiverse countries, such as Brazil (Mittermeier et al., 2005), have underutilized biological resources embedded in a microbial diversity that is poorly studied (Pylro et al., 2014). This diversity has immeasurable societal value (Bodelier, 2011), but the paucity of taxonomic knowledge on microbial species hinders bioprospection projects (Paterson and Lima, 2017), ultimately affecting biotechnology, conservation ecology, medicine, and public health (Hawksworth, 1991). The scarcity of specialized microbial culture collections, particularly in hot spot areas (Lourenço and Vieira, 2004), makes microbial surveys a daunting, but necessary task.

Culture collections identify, catalog, store, and supply microorganisms to end users (Simões et al., 2016). Through those activities, they train scientists and shape the development of microbial taxonomy. Historically, fungal taxonomy and identification have been based mainly on morphological traits (Guarro et al., 1999). However, morphology proved to be insufficient given intraspecific variation and interspecific similarities (Geiser et al., 2007). A polyphasic approach using as many traits as possible seemed to be the best alternative, as the combination of diverse characters could provide a better representation of similarities and robust identifications (Samson and Varga, 2009). Biochemical and physiological characters, such as secondary metabolites and growth profiles, were used, followed by molecular data from multiple housekeeping genes, such as ITS, calmodulin, and beta-tubulin (Frisvad et al., 2007). In the new era of spectral techniques in microbial identification using the matrix-assisted laser desorption/ionization time-of-flight mass spectrometry (MALDI-TOF MS), mycologists have added spectral data to their polyphasic approach (Lima and Santos, 2017). MALDI-TOF MS proved to be a suitable method to identify fungi as it generates species-specific spectral data of large organic molecules, such as proteins (Santos et al., 2010). Santos et al. (2017) and Lima and Santos (2017) have described MALDI-TOF MS' basic principles that can be summarized as follows: the fungal sample is covered with an organic matrix, which functions as an energy mediator, and then subjected to a pulsed laser. When the laser shuts the sample, the matrix mixture generates a gas-phase ions plume.The ions will fly separately according to their ionic mass and eventually they will reach the detector.

Rapidly, MALDI-TOF MS revolutionized clinical microbiology and streamlined the polyphasic approach as being accurate, rapid, and cost-effective. The technique has been successfully applied in the identification of filamentous fungi (Santos et al., 2010), yeasts (Lima-Neto et al., 2014), bacteria (Rodrigues et al., 2014), and viruses (Calderaro et al., 2014). Yet, MALDI-TOF MS has limited capacity in identifying closely related fungal taxa, such as the dimorphic fungi with mycelial-to-yeast phase transitions or highly encapsulated yeasts (Lima and Santos, 2017). Another drawback is the quality and the extension of spectra from microbial taxa that each database delivers (Santos et al., 2017).

## Fungal Community Ecology Using MALDI-tOF Ms Requires Collaborative Efforts Toward Curated Mass Spectral Databases

Traditional polyphasic identifications may not always be appropriate for strictly clinical settings, because they are time-consuming and onerous. Taking a long time to identify a pathogen can ultimately cost the life of patients (Brown et al., 2012). That is why rapid and accurate methods such as MALDI-TOF MS (Alanio et al., 2011) or sequence-based analyses (Balajee et al., 2009) are attractive. Conversely, microbial surveys in ecological studies should aim to identify and characterize microorganisms in a more complete manner (Hanemaaijer et al., 2015). A polyphasic approach is therefore suitable, as it not only reduces misidentifications, but it also gives a more holistic picture of the organisms sampled (Samson and Varga, 2009).

MALDI-TOF MS has potential use in microbial ecology studies (Santos et al., 2016) given adequate data handling. The main obstacle is the lack of reference databases for non-medical strains (Rahi et al., 2016). Every single study on MALDI-TOF MS species identification points to the importance of reference databases, as sample preparation methods, matrix components, and even type of material analyzed (either whole cell or supernatant) may influence the quality and accuracy of spectra (Santos et al., 2017). Accordingly, databases need standardization for as many microbial groups as possible.

As different taxa can demand different protocols, generating new reference spectra should be a cooperative work among different laboratories to generate standardized (and comparable) public databases (Sauget et al., 2017). Other public databases, such as the National Center for Biotechnology Information–Sequence Read Archive (NCBI-SRA), can provide excellent material for comparative studies (Sanitá Lima and Smith, 2017a,b), because they are teeming with high quality genomic and transcriptomic data (Smith and Sanitá Lima, 2016). Hitherto, gene and protein databases are also crammed with poorly annotated sequences and datasets (Sanitá Lima and Smith, 2017c), so their spectral counterpart should avoid running into the same problem.

## Challenges of Studying Eukaryotic Microbial Communities

Characterizing and identifying the constituents of microbial assemblages unravel surprising ways microorganisms affect ecosystems and human activities (Peay et al., 2016). For instance, belowground microbial decomposer communities respond to ecosystem engineers in Boreal peatland (Palozzi and Lindo, 2017a) suggesting local adaptation to plant litter nutrients (Palozzi and Lindo, 2017b). Microbial co-cultures also produce synergistic enzymatic mixtures widely used in industrial fermentative processes (Lima et al., 2016). Yet, the diversity of microbial communities is mostly unknown, particularly in megadiverse countries (Scheffers et al., 2012). The “meta-omics” approach, namely metagenomics, metatranscriptomics, metaproteomics, and metabolomics, changed our understanding of microbial communities (Jansson and Baker, 2016), mainly for prokaryotes. Eukaryotic microorganisms impose greater challenges to community-level studies because their genomes do not robustly predict their ecological roles as in bacteria (Keeling and del Campo, 2017). Traditional transcriptomics and the more recent approach of single-cell genomics/transcriptomics can aid in the characterization of eukaryotic microbial communities (Kolisko et al., 2014), but better understanding the ecology of eukaryotic microbes will only be possible if organisms are isolated, cultured, and studied at the cellular level (Keeling and del Campo, 2017). Reiterative pipelines of phylogenomics and sub-culturing studies can then help to disentangle microbial communities (Cibrián-Jaramillo and Barona-Gómez, 2016) facilitating their final identification through MALDI-TOF MS, for instance.

Estimates on the number of fungi species vary considerably and even as many as 1.5 million species seems to be a conservative number (Hawksworth and Lücking, 2017). Fungi are everywhere, from the bottom of the oceans (Richards et al., 2012) to the alpine glaciers (Brunner et al., 2011). Identifying these fungal communities will then shape our understanding of evolution, ecosystems services, and biogeochemical cycles as well as influence human progress (Hawksworth, 2009). Given the dimension of fungi diversity, the demand for skilled personnel is high (Hibbett and Taylor, 2013). Indeed, the deluge of genomic and transcriptomic data from all sorts of organisms, requires traditional taxonomists like never before and calls back the old-school naturalist approach to Biology (Keeling and del Campo, 2017). Culture collections together with their broader counterpart, microbial Biological Resource Centers (mBRCs), will play fundamental roles in this process, as they are hubs for taxonomic training and long-term preservation of microorganisms. Standardized identification methods and catalogs of microbial strains (Stackebrandt and Smith, 2018) will assist microbial ecology, whereas MALDI-TOF MS has the potential to become a unifying method of identification. However, integration among laboratories to standardize protocols and to improve databases is the main bottleneck.

## Author Contributions

NL, CS, and MP proposed and conducted the discussions that led to this opinion piece. MS and RC researched the literature and drafted the manuscript. NL, CS, and MP revised the manuscript. All authors approved the final version.

### Conflict of Interest Statement

The authors declare that the research was conducted in the absence of any commercial or financial relationships that could be construed as a potential conflict of interest.
